# The Effect of Anlotinib Combined with anti-PD-1 in the Treatment of Gastric Cancer

**DOI:** 10.3389/fsurg.2022.895982

**Published:** 2022-04-12

**Authors:** Wubin Zheng, Guoqiang Sun, Zhitao Li, Fan Wu, Guangshun Sun, Hongyong Cao, Jin Zhou, Yong Ma

**Affiliations:** Department of General Surgery, Nanjing First Hospital, Nanjing Medical University, Nanjing, Jiangsu, China

**Keywords:** anlotinib, gastric cancer, PD1, immunotherapy, VEGFR - vascular endothelial growth factor receptor

## Abstract

**Background:**

Protein tyrosine kinase (PTK) signaling pathway has been confirmed to be involved in the proliferation, differentiation and migration of tumor cells. Anlotinib, as a multi-target tyrosine kinase inhibitor, which can inhibit the expression of vascular endothelial growth factor receptor (VEGFR), has been confirmed to have significant therapeutic effects on non-small cell lung cancer, medullary thyroid carcinoma, and soft tissue sarcoma, but the therapeutic effect on gastric cancer (GC) is still unclear.

**Methods:**

Anlotinib was screened out of 880 drugs through Cell Counting Kit 8 (CCK-8) technology. TCGA was used to detect the expression of VEGFR in GC, and Kaplan-Meier Plotter was used to analyze the correlation between the expression of VEGFR and the survival rate of GC patients. The impacts exerted by anlotinib to GC cell proliferating, migrating and invading processes were assessed through wound healing assay, transwell assay, and proliferation assay in vitro. In vivo experiments of GC were performed in C57/B6 mouse model to evaluate the function of anlotinib and PD-1 antibody.

**Results:**

It was found from more than compunds that anlotinib has a significant inhibitory effect on GC cells. In vitro experiments show that anlotinib can significantly inhibit the proliferation, invasion and proliferation of GC cells. The expression level of VEGFR is related to the prognosis and survival of GC. GC patients with low expression of VEGFR have better survival. Anlotinib can inhibit the expression of PD-L1, and achieve better therapeutic effects after combined with PD-1 antibody.

**Conclusion:**

The present study reveals that anlotinib down regulates PD-L1. The combination of anlotinib and PD-1 monoclonal antibody is beneficial to GC therapy.

## Introduction

Gastric cancer (GC) is one of the most common malignant tumors worldwide, with the 5th highest incidence rate and the 3rd highest mortality rate among all malignant tumors, after lung cancer and liver cancer (1). Due to the insidious symptoms of GC, patients are often diagnosed with progressive or even advanced GC, and the survival period is extremely low. With the rapid development of treatment technology in recent years, the survival rate of patients with progressive gastric cancer is only about 20% (2). To date, surgery is still the main treatment modality for GC, while for those without surgical indications or who need to be supplemented with chemotherapy before and after surgery. The current first-line chemotherapy for gastric cancer is fluorouracil in combination with platinum ± epirubicin/docetaxel/irinotecan as recommended by the National Comprehensive Cancer Network (NCCN) in 2016. Nevertheless, the survival rate of GC patients is still extremely low, indicating that finding new and effective chemotherapeutic drugs to improve the treatment effect and prolong the survival rate of GC patients is still the top priority of gastric cancer treatment at present. In recent years, molecular targeted drugs have become a popular research topic because of their high efficiency and low toxicity in treating cancers such as breast, colorectal, thyroid, and non-small cell lung cancer. At the same time, TKIs plus immunotherapy have gradually been widely used in gastric cancer and other malignant tumors, and have received good feedback (3–5).

Tumor metastasis (especially peritoneum and liver) and hypoproteinemia are important factors affecting the survival of GC patients. The protein tyrosine kinase (PTK) signaling pathway is involved in the proliferation, differentiation and migration of tumor cells. Interfering or blocking the PTK pathway can stop the growth of tumor cells and thus achieve therapeutic effects (6). Anrotinib is a multi-target tyrosine kinase inhibitor, an orally administered small molecule drug that exerts an inhibitory effect on tumor angiogenesis and tumor cell proliferation by selectively inhibiting vascular endothelial growth factor receptor (VEGFR), fibroblast growth factor receptor (FGFR) and platelet-derived growth factor receptor (PDGFR) (7). Current researches have demonstrated that anlotinib has significant therapeutic effects on non-small cell lung cancer, medullary thyroid cancer, and soft tissue sarcoma, significantly prolonging overall survival (OS) and progression-free survival (PFS) with the adverse effects were small (8–10). However, there is still a lack of evidence for the efficacy of this treatment in GC.

GC evades the immune system by down-regulating tumor expression, up-regulating immune checkpoints, inactivating cytotoxic T cells and altering the tumor immune microenvironment. Anti-tumor therapies that inhibit negative immunoregulatory mechanisms, namely immune check-point blockade (ICB), have become a hot topic of current research. The application of PD-1(programmed death 1)/PD-L1(programmed death ligand 1) inhibitors to block the immune checkpoint is the most promising ICB therapy (11, 12). It has been reported that PD-1 /PD-L1 expression is detected abnormally in GC patients and is closely related to tumor progression and patient prognosis. The effectiveness of anlotinib and PD-1 antibody in the treatment of GC is currently unclear, and further studies are needed to provide guidance for the clinical treatment of GC.

## Materials and Methods

### Cell Cultures

Human GC cell lines (AGS, MKN-45) were provided by Shanghai Institutes for Biological Sciences in China. All cells were grown in a humidified incubator at 37°C with 5% CO2 in RPMI-1640 medium (Biological Industries, Israel) containing 10% fetal bovine serum (Gibco, Austria) and 1% penicillin-streptomycin.

### Quantitative Reverse Transcription Polymerase Reaction (qRT-PCR)

Total RNAs from cells were extracted using TRIzol reagent (Invitrogen, USA) according to the manufacturer’s instructions. Overall RNAs were reverse transcribed to cDNA by a reverse transcription kit (Takara, Japan). We quantified PD-L1 mRNA and performed PCR assays based on the SYBR Green PCR kit (Takara, Japan) and based on a SYBR Green PCR Kit (RiboBio, China). Before calculating, the levels of mRNA expression status were normalized with GAPDH.

### VEGFR Expression Level Analysis and Clinicopathological Analysis

The expression of VEGFR in GC tissues and corresponding normal tissues was investigated using TCGAportal. VEGFR expression was compared in GC patients of various types, stages, grades, and nodal metastasis status using UALCAN. Kaplan-Meier Plotter was used to compare correlations between VEGFR expression and overall survival (OS), post-progression survival (PPS), and first progression survival (FP)**.** Tumor Immune Single-cell Hub (TISCH) was used to provide detailed cell-type annotation at the single-cell level, enabling the exploration of TME across different cancer types including GC.

### Tools for VEGFR Location in Cells

The Human Protein Atlas, compiling numerous reports and tissue, cell and pathology atlas forms, and gene data in cells and tissues, was utilized for obtaining VEGFR location in cells.

### Tool for Immune-Related Analysis of VEGFR

TISIDB was adopted for delving into the spearman correlations between VEGFR and immune-modulator expression.

### Cell Counting Kit-8 Proliferation Assay

To detect the inhibitory effect of drugs on cell proliferation, researchers used the CCK-8 kit (Ribobio, China). Drug Screening: GC cells (2,000/well) were spread in 96-well plates and cultured for 24 h. The drug to be screened was added to each well at a concentration of 5 µM. After a 24-hour incubation period in a humidified incubator at 37°C with 5% CO2, 10 µl of CCK-8 reagent was added to each well and the incubation was continued for 2 h before the absorbance at 450 nm was measured using an enzyme marker. Selleck (USA) provided the drug to be screened as well as the anlotinib used in the assay.

### Wound Healing Assay

After the fusion of both AGS or MKN-45 cell lines reached 90%, artificial linear incisions were created with a standard 200 µl pipette tip, washed twice with PBS to remove any free-floating cells and debris, and replaced with new medium. 5 µM and 10 µM of anlotinib were added to the medium and equal concentrations of dimethyl sulfoxide (DMSO) were added to the control group. The plates were incubated at 37°C with 5% CO2 in a humidified incubator. At different time points (0 h, 24 h, 48 h), the wound healing within the scratch line was observed, and representative scratch lines were photographed.

### Transwell Assay

Anlotinib was added to the GC cell lines after they had reached 90% fusion, and equal concentrations of dimethyl sulfoxide (DMSO) were added to the control group. The plates were then placed in a humidified incubator at 37°C with 5% CO2 for 24 h. Complete the inoculation of GC cell lines according to the manufacturer’s instructions. The upper chamber was added with 200 µl RPMI-1640 medium and 4×10^6^ GC cells (with or without treatment with anlotinib) and the lower chamber was added with 700 µl RPMI-1640 medium containing 10% fetal bovine serum as a chemotactic agent. The upper chamber was fixed with formaldehyde and stained with 0.1% crystal violet for 15 min after 24 h of culture. Finally, under an inverted microscope, the treated cell lines were photographed and counted.

### Colony Formation Assay

AGS and MKN-45 cell lines were spread into six-well plates at a density of 1,000 cells/well, and they were treated with anlotinib, respectively, and equal concentrations of DMSO were added to the control group. The six-well plates were incubated for 14 days at 37°C with 5% CO2 in a humidified incubator. The cell colonies were fixed using formaldehyde and stained with 0.1% crystal violet for 15 min. The colonies were photographed and counted. All assays were carried out in triplicate.

### Western Blotting

Abcam Corporation (UK) provided the anti-PD-L1 antibody. GADPH was obtained by lysing cells in RIPA lysis buffer (RIPA, Beyotime, China). The protrin blocked using 5% skim milk powder received the incubation using primary antibody, anti-GADPH and anti-PD-L1, at 4°C for 12 h. After that, the synthesized membranes were incubated for 2 h with a secondary antibody (1:5,000). Lastly, with enhanced chemiluminescence kit (Pierce, USA). Image J was using to analyze the images of western blot, and the quantitative expression level of proteins were examined as the band-intensity.

### Mice Model

The animal experiment was approved by Nanjing Medical University’s animal management committee, and all experiment procedures and animal caring conformed to the institutional ethics directions for animals-related experimental processes. For GC study, we conducted the random separation of 20 male C57/B6 mice aged 5 weeks in anlotinib, PD-1 monoclonal antibody (BP0273, bioxcell, USA), anlotinib+ PD-1 monoclonal antibody, PBS (*n* = 5 for the respective group) in a range of MFC cells. In the mice inguen, MFC cells were injected subcutaneously. The researchers killed all the mice in the experiment, collected tumor sizes and weighed tumors for further experimental tests.

### Statistical Analysis

We compared continuous information based on an individual *t*-experiment in the two groups.We carried out the analyses largely based on Graphpad Prism 8.0 (USA), and *p*-value <0.05 was reported with statistics-related significance.

## Results

### Comprehensive High-Throughput Screening Shows that Anlotinib has a Significant Inhibitory Effect on GC Cells

**Figure 1:**
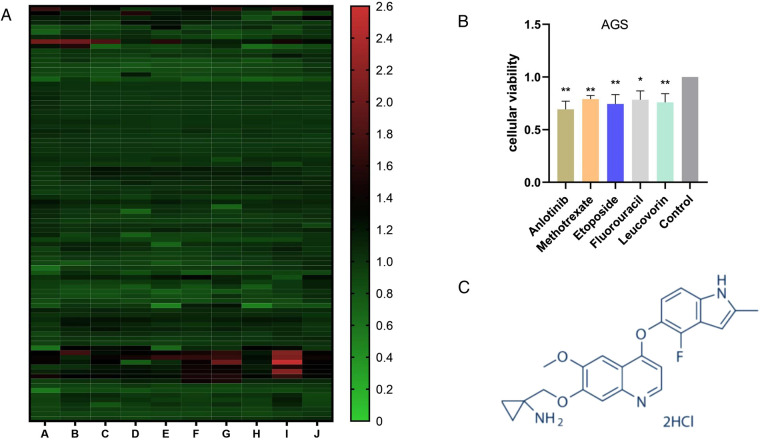
Screening of the inhibitory effects of multiple drugs on GC cells: (**A**) The proliferation of more than 880 drugs after treatment of GC cells. (**B**) Compared with the four drugs commonly used in GC chemotherapy, Anlotinib has a more obvious inhibitory effect on the proliferation of GC cells. (**C**) Anlotinib compound structure. (Picture from www.selleck.cn). *P < 0.05; **P < 0.01.

We used CCK-8 experiment to detect the cell proliferation efficiency of 880 kinds of drugs after drug treatment, and the experimental results found that anlotinib has obviously inhibitory effect on GC cell proliferation (Figure 1A). After the initial screening was completed, we used the same experiment to compare anlotinib with traditional gastric cancer chemotherapy drugs such as Methotrexate, Etoposide, Fluorouracil and Leucovorin. The cell proliferation results indicated that anlotinib’s inhibitory effect on GC cells is better than traditional drugs (Figure 1B). The structure of anlotinib is shown in the Figure 1C, and we decided to study the role of anlotinib in the treatment of GC.

### VEGFR is Significantly Correlated with Clinical Prognosis

To further explore the prognostic potential of VEGFR (the target of anlotinib) in GC, TCGA portal and Kaplan-Meier Plotter were used.

**Figure 2:**
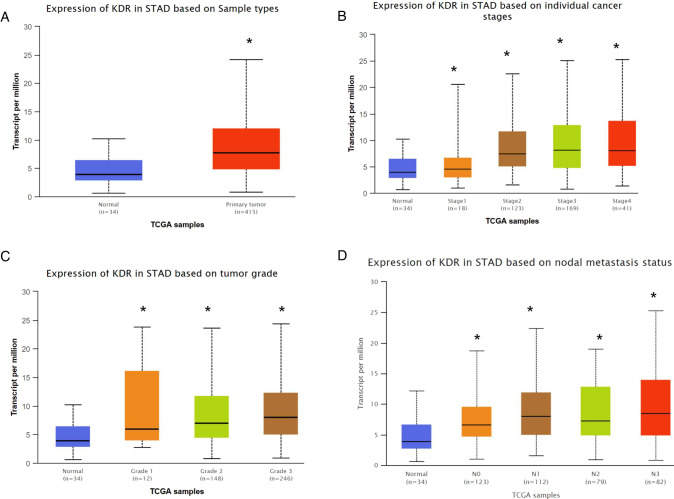
VEGFR expression in GC. (**A**) VEGFR expression based on sample type. (**B**) VEGFR expression based on individual cancer stages. (**C**) VEGFR expression based on tumor grade. (**D**) VEGFR expression based on nodal metastasis status. *p < 0.05.

**Figure 3:**
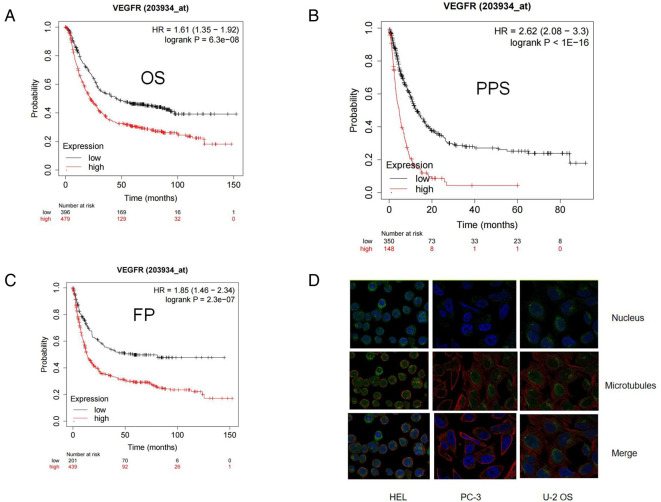
High expression of VEGFR indicated a worse GC clinical prognosis from Kaplan Meier Plotter. (**A**-**C**) Kaplan Meier survival curve showed the relationship between PARP and overall survival (OS), post-progression survival (PPS), and first progression survival (FP) in GC patients. (**D**) The location of VEGFR in cell.

The TCGA portal showed that the expression of VEGFR in tumor tissues was obvious higher than that in normal tissues (Figure 2A). The expression level of VEGFR in different stages, tumor grades,or lymph node metastasis of individual cancer indicated that the expression level of VEGFR in GC tissues was also higher than normal tissues (Figures 2B–D). The prognostic potential of VEGFR in GC was further examined using Kaplan-Meier Plotter. The results showed that GC patients with higher VEGFR expression had lower overall survival (OS), post-progression survival (PPS), and first progression survival (FP) (Figures 3A–C).

### Research Results of VEGFR at Single Cell Level

**Figure 4:**
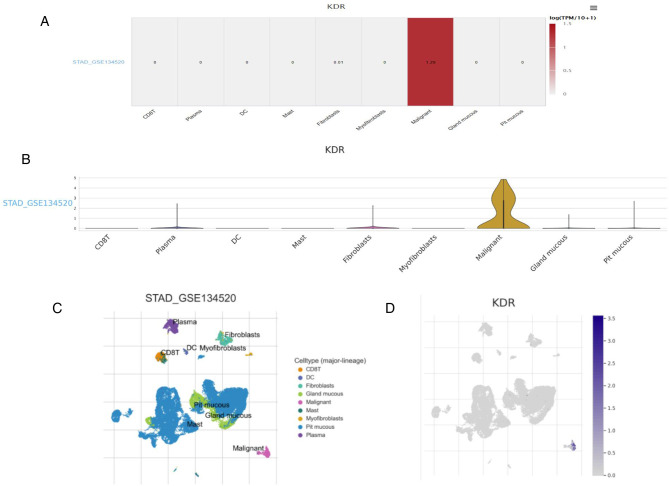
Research results of VEGFR at single cell level. (**A**) Heatmap displays the value of VEGFR expression in different cells from database. (**B**) Violin diagram displays the distribution of VEGFR expression in different cells from database. (**C,D**) Single-cell cluster map of VEGFR in different databases.

We studied the expression of VEGFR at the single cell level. Figures 4A,B showed the distribution of VEGFR expression in GC database. In database GSE134520, the VEGFR was enriched at the malignant, fibroblasts, and plasma cell clusters (Figures 4C,D). These results suggest that VEGFR mainly function in malignant other than other cells. Moreover,according to the human protein atlas database, VEGFR is located in the cell membrane in cells (Figure 3D).

### Anlotinib Inhibits the Proliferation, Invasion and Migration of GC Cells

**Figure 5:**
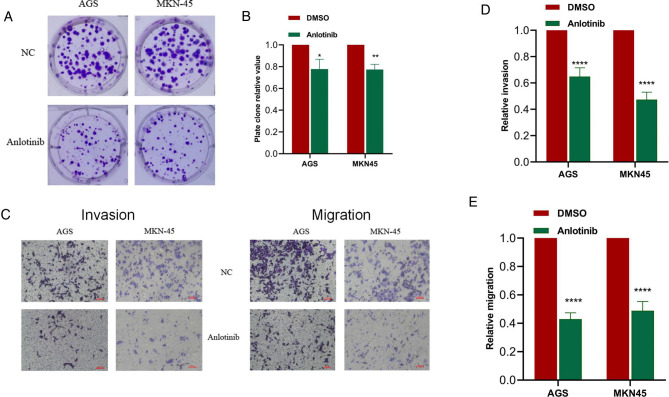
Anlotinib can significantly inhibit the proliferation, migration and invasion of GC cells. (**A,B**) Plate cloning assays proved that Anlotinib can significantly inhibit the proliferation of GC cells. (**C**-**E**) The results of transwell migration and invasion assays proved that Anlotinib can inhibit the migration and invasion of GC cells. *P < 0.05; **P < 0.01; ****P < 0.0001.

**Figure 6:**
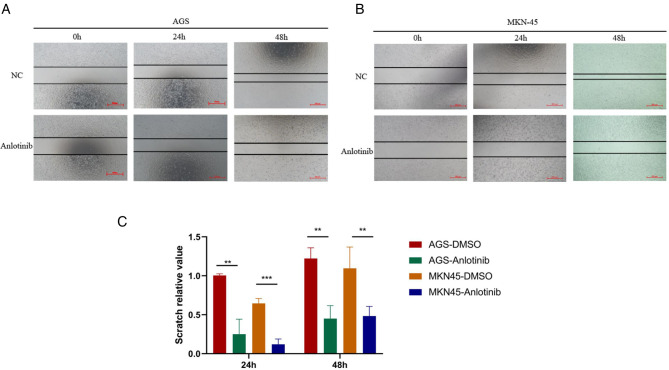
Scratch test results of Anlotinib in GC cells. (**A–C**) Scratch assay proved that Anlotinib can inhibit the migration of GC cells. *P < 0.05; **P < 0.01; ***P < 0.001.

For gaining insights into the effects exerted by anlotinib on the biological behaviors of GC cells, we used anlotinib to treat GC cell lines including AGS and MKN-45 respectively. The results of plate cloning experiments show that anlotinib can inhibit the proliferation of GC cells (Figures 3A,B). The transwell assay revealed that anlotinib can significantly inhibit the invasion and migration potential of GC cells, compared with the untreated group (Figures 5C–E). At the same time, wound healing assay once again confirmed that in GC cell lines, the wound healing rate of GC cells treated with anlotinib was significantly lower than the control group (Figures 6A–C).

### VEGFR Expression was Correlated with Immune Factors

**Figure 7:**
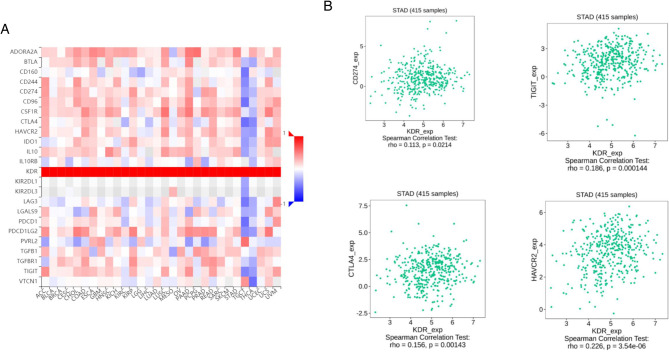
Correlation between VEGFR expression and immunoinhibitors in GC. (**A**) The heat map shows the correlation between VEGFR and immunosuppressive factors in different cancers. (**B**) Line graph shows the correlation of VEGFR with specific immune indicators in GC.

Existing studies have confirmed that the immune system is closely related to the occurrence and development of tumors. Therefore, we studied the relationship between the expression of VEGFR and immune factors. As shown in Figures 7A,B, there was a strong positive correlation between the expression of immunoinhibitors (CD274,TIGIT,CTLA4,HAVCR2) and the expression of VEGFR.

### The Combined use of Anlotinib and anti-PD-1 has a Significant Inhibitory Effect on GC Cells

Next, we needed to explore whether the effect of anlotinib on the expression of PD-L1 in GC cells is consistent with the above results.

**Figure 8:**
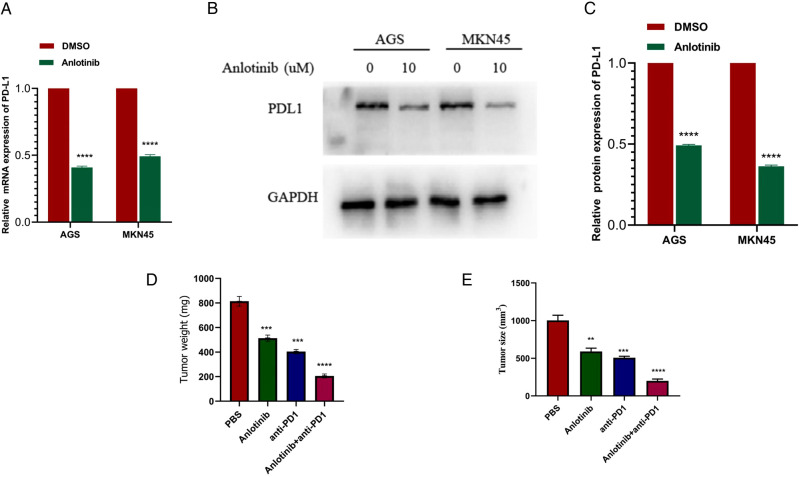
Anlotinib combined with anti-PD-1 has a significant therapeutic effect on GC. (**A**) QRT- PCR results show that Anlotinib can significantly reduce the expression of PD-L1. (**B,C**) Western blotting experiment proved that Anlotinib can significantly reduce PD-L1 expression. (**D**) The relative weights of tumors were evaluated in C57BL/6 mice. (**E**) The relative tumor sizes were evaluated in C57BL/6 mice. ***P < 0.001; ****P < 0.0001.

QRT-PCR results showed that the expression of PD-L1 in GC cells treated with anlotinib was significantly lower than that in the control group (Figure 8A). Western blotting analysis results from the protein expression level suggested that anlotinib can significantly reduce the expression of PD-L1 relative to the control group (Figures 8B,C). To examine the association between anlotinib and the growth of GC in vivo, we injected MFC cells into subcutaneous groin of C57/B6 mice, and then we administered intraperitoneally anlotinib or PD-1 antibody alone or in combination to mice and analyzed tumor growth in 20 days, as well as weight of tumors after the mice were killed. As indicated from the results, the combination group of anlotinib combined with PD-1 monoclonal antibody has a better inhibitory effect on tumors, compared with the single-drug group and the control group (Figures 8D,E).

## Discussion

Although in the past few decades, the preoperative or postoperative chemotherapy regimen for GC has gradually been determined as fluorouracil combined with platinum ± epirubicin/docetaxel/irinotecan, but the current five-year survival rate of GC patients is still low, and the five-year survival rate for advanced GC patients with tumor metastasis is lower. Therefore, researchers are still looking for better new and effective chemotherapeutics that can prolong the survival rate of patients and improve the treatment effect.

In the past five years, ICB therapy has revolutionized the treatment of some solid tumors or hematological tumors, and there are still multiple new studies, hoping that ICB therapy can be more widely used in other tumor treatment areas in the future (4, 5). TKIs combined with immunotherapy is a brand-new weapon for tumor treatment in recent years. This method has not only been used in the treatment of GC, but also widely used in other tumors, and the therapeutic effect has been gradually verified in the laboratory and clinical practice.

Anlotinib is a multi-target tyrosine kinase inhibitor, an oral small molecule drug that selectively inhibits vascular endothelial growth factor receptor, fibroblast growth factor receptor, platelet-derived growth factor receptor. It plays an important role in inhibiting tumor angiogenesis and tumor cell proliferation. Studies have shown that the antiangiogenic effect of anlotinib is comparable to Sunitinib and stronger than that of Sorafenib (13). In the past, anlotinib was only used in the fields of non-small cell lung cancer (14), medullary thyroid carcinoma (15), soft tissue sarcoma (16), and it has a good therapeutic effect. However, the effect of anlotinib on GC is still lacking researching. Immunotherapy is the current research hotspot in the field of tumor treatment. ICB therapy, especially PD-1/PD-L1 inhibitors, is the most promising treatment method among them. Carrilizumab is a classic PD-1 inhibitor that has been frequently used in clinical practice recently. A large part of patients with gastric cancer can detect the abnormal expression of PD-1/PD-L1, so the therapeutic effect of carrilizumab in GC is worthwhile expect. Li et al. (17) found that antiangiogenic drugs also play a regulatory role in suppressing immune signals, including the expression of PD-1 in tumor infiltrating T cells. In addition, several studies have suggested that antiangiogenic agents may increase the efficacy of immune checkpoint inhibitors, and the combination of two pathway inhibitors is often feasible (18, 19).

Our research first found that the inhibitory effect of anlotinib on GC cells is not inferior to that of classic GC chemotherapy drugs through reasonable screening of a large number of drugs. Then, we used the TCGA public database to analyze and found that the low expression of VEGFR can significantly improve the prognosis of GC and prolong survival. In addition, according to Kaplan-Meier univariate survival analysis, we found that the OS, FP, and PPS of GC patients with low VEGFR expression were significantly longer than those with high VEGFR expression. The results of in vitro experiments can prove that anlotinib can significantly inhibit the proliferation and invasion of GC cells, and it is obviously concentration-dependent. The results of tumor formation experiments in mice prove that anlotinib has a significant therapeutic effect on tumor tissues, and it can significantly enhance the efficacy when used in combination with PD-1 monoclonal antibody.

However, our study of anlotinib still has certain limitations. First, we only found that anlotinib has a therapeutic effect on GC, but did not further explore the mechanism of the inhibitory effect. At the same time, the GC cell lines we selected in the cell experiment validation did not include HER2 negative, which cannot explain the therapeutic effect of anlotinib on HER2 negative GC. These will be reflected in the team’s future research.

In conclusion, our study found that anlotinib, as a multi-target tyrosine kinase inhibitor, can significantly inhibit the proliferation and invasion of GC cells, and has a significant therapeutic effect on GC tumor tissues. Moreover, when used in combination with PD-1 inhibitors, anlotinib can significantly improve the efficacy. Our research may provide a new drug choice for the chemotherapy of GC.

## Data Availability

The datasets presented in this study can be found in online repositories. The names of the repository/repositories and accession number(s) can be found in the article/supplementary material.
